# The Visit of the Medical Editors

**Published:** 1894-03

**Authors:** 


### THE VISIT OF THE MEDICAL EDITORS.

We were pleased to receive a visit on the 17th ultimo from the editors of some of our esteemed Northern contemporaries. This editorial party was making a short and rapid tour through certain portions of the South for the purpose of investigating the climatic and sanitary conditions of those sections. Their investigations related especially to the determination of the most favorable locations for the climatic and hygienic treatment of consumption. The party consisted of Dr. W. C. Wile, editor of the New England *Medical Monthly*, Danbury, Connecticut; Dr. Ferdinand King, of the *Polyclinic*, New York; Hon. Clark Bell, editor ot *Medico-Legal Journal*, New York; Dr. Howard Van Rensselaer, editor *Medical Annals*, Albany; Dr. W. Blair Stewart, editor of *Medical Bulletin*, Philadelphia; Dr. Angell, of New York; Dr. Stewart, of New York; Mr. Stewart Griffing, representing the Connecticut press; and Dr. L. J. Picot, Littleton, N. C. The Rev. Dr. McNeil, of New York, was present also to conserve the morals of the party.

These visiting gentlemen were well received and entertained in the several places in North Carolina and Georgia where they stopped, and expressed themselves everywhere as pleased with the Southern hospitality shown them. In Atlanta they were met by a committee from the Atlanta Society of Medicine and escorted to the rooms of the Commercial Club. Here a reception awaited them, and opportunity was given to meet the local medical profession and the leading representatives of the Club. The next day they were driven over the city.

We are glad to receive such visits from our Northern friends. From a climatic point of view we are sure that our section can fulfill all the conditions which they sought. We wish our colleagues from the North would see more of us and know us better. They should come again, therefore, when they can stay longer.

---

Rev. J. B. Hawthorne, of this city, said in his sermon, February 18th: "*If all the thieves were put into the chain-gang to-morrow, . . . it would shut the doors of real estate offices and thin the ranks of the legal and medical fraternities.*" In regard to the real estate business, Dr. Hawthorne probably speaks by the card, because he has been interested in some land schemes himself in a quiet way as a "side line" to the sacred ministry. He therefore knows the tricks of the trade. We do not know what motive or experience prompted the good doctor in his stricture upon the medical fraternity. The only relation that we know of which he has sustained toward the medical profession has been to receive free medical attention for himself and family whenever occasion required. Such insinuations, therefore, as the above come with very poor grace and savor of the meanest ingratitude. And all of this, too, from a man, a minister, who owns, or did own, a large part of the stock in a patent medicine humbug, King's Royal Germeteur, which consists only of the addition of one pint of hydrochloric acid, costing twenty cents, to a barrel of water, costing nothing, the mixture selling for one dollar a quart! The doctor ironically selected for his text that morning: *He that is without sin among you, let him cast the first stone.* We think that this great evangel of all that is good and honest, who poses as the public censor, might make a personal application of his text with considerable advantage.

---

DR. ARCHER ATKINSON, of Baltimore, contributes to the Virginia *Medical Monthly* for February an article on Longevity, and gives a list of persons known to have lived one hundred years and over. This list shows a careful collection from many sources, not now to be had, of more than two hundred and fifty names of centenarians. It is a curiosity and might serve in helping to verify the longevity of certain families. It shows that no one method of life, no one profession or trade and no special locality favors the prolongation of life.

---

A NEW remedy which may supplant atropine as a mydriatic is the hydrobromate of scapolamine. It is said to have all the advantages of atropine as a dilator of the pupil, but does not increase intraocular pressure. It is applied to the eye in solution, one grain to the ounce of water.

